# High-Resolution Reconstruction of Temperature Fields Based on Improved ResNet18

**DOI:** 10.3390/s24206564

**Published:** 2024-10-12

**Authors:** Leilei Ma, Jungang Ma, Manlidan Zelminbek, Wenjun Zhang

**Affiliations:** Xinjiang Uygur Autonomous Region Research Institute Of Measurement & Testing, Urumqi 830011, China; maleishiyuan@126.com (L.M.); manlidan2011@163.com (M.Z.); taozhiyaoyaowen@163.com (W.Z.)

**Keywords:** temperature field reconstruction, deep learning, multi-scale feature aggregation, mean square error

## Abstract

High-precision measurement of temperature value distributions in production scenarios is of great significance for industrial production, but traditional temperature field reconstruction algorithms rely on the design of manual feature extraction methods with high computational complexity and poor generalization ability. In this paper, we propose a high-precision temperature field reconstruction algorithm based on deep learning, using an efficient adaptive feature extraction method for temperature field reconstruction. We design an improved temperature field reconstruction algorithm based on the ResNet18 neural network; introduce the CBAM attention mechanism in the model; and design a feature pyramid, using M-FPN, a multi-scale feature aggregation network fusing PAN and FPN, to make the extracted feature information propagate multi-dimensionally among different layers to improve the feature characterization ability. Finally, the mean square error is used to guide the model to optimize the training so that the model pays more attention to the data and reduces the large error to ensure that the gap between the predicted value and the real value is small. The experimental results show that the reconstruction accuracy of the improved algorithm presented in this paper is significantly better than that of the original algorithm in the case of typical peaked temperature field distributions.

## 1. Introduction

Highly accurate temperature field reconstruction can restore the precise temperature distribution in the industrial production environment, and the precise temperature value is an important parameter to guide industrial production [1,2,3]. In thermochemical processes, accurate reconstruction of the temperature field is essential to optimize the temperature distribution within the reactor. By accurately controlling the reaction temperature, thermal conversion efficiency can be improved, energy consumption can be reduced, and by-product generation can be reduced. In the energy sector, temperature field monitoring can help improve the efficiency of thermoelectric conversion devices, such as in coal-fired power plants, where accurate temperature field data can help optimize the combustion process within the boiler, improve thermal conversion efficiency, and reduce emissions. In addition, in environmental engineering, e.g., waste-to-energy incineration, temperature field reconstruction can also be used to monitor and optimize the combustion process, ensuring maximum energy utilization and reducing harmful emissions. Through these specific applications, temperature field reconstruction technology can be used to promote energy saving and emission reduction, improve the efficiency of energy use, and at the same time realize the fine management of the thermal conversion process, which is of great significance to the sustainable development of enterprises.

Existing temperature field measurement methods are mainly categorized into two forms: contact measurement and non-contact measurement. Common contact sensors include thermocouples [4], thermistors [5], etc. However, since they need to be in contact with the object to be measured, they may cause damage to the object to be measured or affect its surface properties. In addition, contact sensors cannot be used to measure the temperature distribution of moving or irregularly shaped objects. Non-contact measurement methods include infrared temperature measurement, thermal imaging camera temperature measurement, etc. However, infrared measurement is greatly affected by the environment, which may lead to a reduction in temperature measurement accuracy. Thermal imaging cameras are more costly and require more stringent environmental conditions, such as lower background radiation and less humidity. Among non-contact measurement methods, acoustic tomography (AT) technology has the advantages of non-invasiveness, wide applicability, high resolution, multi-parameter measurement, real-time capability, and environmental adaptability [6].

AT measurement is a method that utilizes sound wave propagation characteristics to measure the internal structure and temperature distribution of an object. It first requires a set of sound sources and receivers to be arranged around or inside the object to be measured. The receivers pick up the signals propagated by the sound waves and convert them into electrical signals. These signals contain the propagation of sound waves at different locations and in different directions. The received acoustic signals are processed, e.g., using inversion algorithms, to infer the structure and temperature distribution inside the object under test based on the propagation path of the acoustic waves and the received signals. In this type of measurement, an accurate and efficient reconstruction algorithm is key to measuring the temperature field distribution [7].

In the process of temperature field reconstruction, traditional temperature field reconstruction algorithms, such as the least-squares QR decomposition algorithm [8], the Tikhonov regularization algorithm [9], and the conjugate gradient method [10], have many limitations. The operational procedure of these methods usually involves the alignment of the observed data with a mathematical model, which may lead to the accumulation of errors, and uncertainties in the model parameters may affect the accuracy of the temperature field. In addition to this, they usually rely on simplifying assumptions or a priori knowledge of the physical system, which may lead to deviations between the model and the actual situation. Most importantly, traditional algorithms are sensitive to the quality and sparsity of the input data, which can lead to poor quality of resolution of the reconstructed temperature field data, thus affecting the accuracy of the temperature field.

In order to overcome the limitations of traditional algorithms in temperature field reconstruction, many scholars have adopted deep learning methods applied in the field of 3D reconstruction inverse problem solving, and AT (inverse problem solving) is one of them. Cai et al. [11] firstly proposed to reconstruct the temperature field using a deep belief network (DBN) and a recurrent neural network (RNN), which demonstrated the advantages of the RNN in terms of noise suppression and the DBN in terms of computational efficiency. Jie Zhang et al. [12], focused on the problems of the large computational volume and the low efficiency of traditional flame temperature field reconstruction for light field imaging, proposed a convolutional neural network (CNN)-based 3D temperature field reconstruction method for flames. The reconstruction time was reduced from 4759 s in the traditional NNLS algorithm to 830 μs in the CNN algorithm. A.T. Sun et al. [13] modeled a long- and short-term memory network combined with a convolutional neural network and used the CNN to extract image features and input them into the LSTN for the reconstruction of the 3D temperature fields of flames and considered the effects of timing noise and image noise. However, traditional convolutional neural network models such as the CNN are highly susceptible to problems such as overfitting and model degradation with the increase in their network layers, resulting in large reconstruction errors [14]. HE et al. [15] proposed Residual Networks (ResNets) in 2016, which overcome the problems of gradient vanishing and gradient exploding by introducing residual connections and jump connections. Among them, ResNet18 is a relatively shallow network structure in the ResNet family with good performance and low computational complexity. However, as the number of network layers increases, it faces some problems, such as the uneven importance of features and the existence of redundant features. In this case, adding an attention mechanism can make the network more focused on key features, effectively improving the network’s expressiveness and generalization ability.

In recent years, regarding the research direction of deep learning in combination with temperature field reconstruction, since generative models such as GANs and VAEs have great advantages in generating realistic data, some scholars have made some innovations by combining their network characteristics. Li, T. et al. [16] investigated the application of GANs in data-driven modeling of thermal convection and presented a case study for the reconstruction of temperature fields; Chen, Y. et al. [17] designed an application of variational auto-encoders (VAEs) in temperature field reconstruction, discussing the training and efficacy of the model; Chen, J. et al. [18] investigated the temperature field reconstruction of GANs in computational fluid dynamic simulation, analyzing the performance of the models and the application scenarios.

However, deep generative models usually require large amounts of computational resources during training; the training process of GANs may be unstable and prone to pattern collapse or non-convergence of training; and though VAEs are able to generate smooth and consistent reconstructions of temperature fields, a generated temperature field may be lacking in detail and limited by the design of the encoder and decoder. Generally speaking, generative models rely on a large amount of high-quality training data for effective learning, and the training process is complex and consumes a lot of computational resources. ResNet18, on the other hand, requires fewer training data for temperature field model reconstruction; the training process is more stable; and because of its simpler architecture and relatively small computational effort, it is more efficient in resource-limited environments.

In this paper, for the original ResNet18 network structure, attention mechanism and feature extraction layer improvement for multi-scale information fusion were carried out.

Regarding the introduction of the attention mechanism, in graphics processing tasks, scholars have been inspired by human visual characteristics and intentionally designed a modeling mechanism that focuses on important local features in a network, i.e., the modeling attention mechanism. Commonly used attention mechanisms include the compression–excitation network (SENet), which adaptively adjusts the feature responses between channels by means of feature recalibration to improve the computational efficiency. Wang et al. [19] proposed a non-local attention network (NLNet), which incorporates global contextual information and has proved its effectiveness in the fields of target detection, image classification, and instance segmentation, though it greatly increases computational loads. Cao et al. [20] proposed the Global Context Network (GCNet) based on Wang et al., which combines the advantages of SENet and NLNet and can effectively improve model performance and generalization ability. In the design of these attention mechanisms, the spatial channel attention mechanism (CBAM) is able to focus on both spatial and channel information of the image with the introduction of relatively few parameters. This multi-scale attention mechanism helps the network to better capture the details and global information in the image, significantly improving the model performance.

Regarding the design of the feature pyramid, the role of the feature pyramid network is to provide a multi-scale feature map to better capture different scale information in tasks such as feature extraction. Higher-level features in a feature pyramid network can contain more abstract and global information, while lower-level features contain more local and detailed information, which allows the network to reuse features at different levels and improve the efficiency of parameter utilization. Tsung-Yi Lin et al. [21] initially designed the FPN (Feature Pyramid Network), and due to its unique network structure with efficient feature reuse, many scholars competed to imitate it and made various improvements based on it. M. T et al. [22] designed BiFPN, which improves the FPN by bi-directional connectivity, better integrates multi-scale features, and improves the network performance. Enzo et al. [23] designed NAS-FPN, which improves the FPN by utilizing neural architectural search techniques to find a feature pyramid structure that is more suitable for the task of target detection and semantic segmentation to improve the performance of the model. Cui et al. [24] designed HRFPN, which is a feature pyramid network for the task of target detection and semantic segmentation using high-resolution images to retain more high-resolution information, introduce additional up-sampling paths to process high-resolution features, and improve network performance. However, these feature extraction network improvements usually bring a large amount of additional parameter volume computation requirements, and the deepening of the network layers also leads to a rise in model training difficulty and an increase in the probability of overfitting.

Therefore, the text incorporates the attention mechanism CBAM and the multi-scale feature fusion pyramid FPN+PAN in the ResNet18 network to improve the model mechanism and structural aspects and enhance the feature extraction performance and problem solving for the temperature field model. The mathematical model of temperature distribution is set up, and the area to be measured is discretized using two profiles of 10 × 10 and 20 × 20; the model then acquires the coarse-grid data and saves the fine-grid temperature distributions. The fine-grid temperature distributions are used as labeled data and combined with the coarse-grid array to form a dataset for training, and the model can be reconstructed with high resolution of the low-resolution temperature field by learning the temperature information corresponding to the labeled data. In this paper, the effectiveness of the two model optimization methods is demonstrated by ablation experiments.

## 2. ResNet18 Network Optimization

Conventional convolutional neural networks (CNNs) usually consist of structures of convolutional, pooling, and fully connected layers, where the convolutional layer is the core component of the CNN and is used to extract features from the input data. The pooling layer is used to reduce the spatial dimensionality of the feature maps, reduce the model parameters and computational effort, and enhance the translational invariance of the model. The activation function is usually applied after the convolutional layer and is used to introduce non-linear properties that enable the network to learn more complex functional relationships. The fully connected layers are usually located in the last layers of the network and are used to map the features extracted from the convolutional layers to the output classes. In contrast, regarding traditional neural networks, due to their structure, the model becomes more likely to memorize the details of the training data than to learn the generic features of the data as the network layers progressively deepen, leading to model overfitting.

Many scholars use Weight Decay, Dropout, and other designs in the model in order to prevent the model from overfitting, but most of these methods will reduce the complexity of the model and to a certain extent will affect the feature extraction ability that the model should have. ResNet innovatively proposes a kind of layer-hopping connection, so that the shallow information of the model is directly connected to the deeper network information in a special structure, which makes it possible to stack the network layers more deeply without the problem of gradient disappearance or explosion. This makes it easier to train deeper networks and has led to breakthrough performance in image recognition tasks.

**Figure 1:**
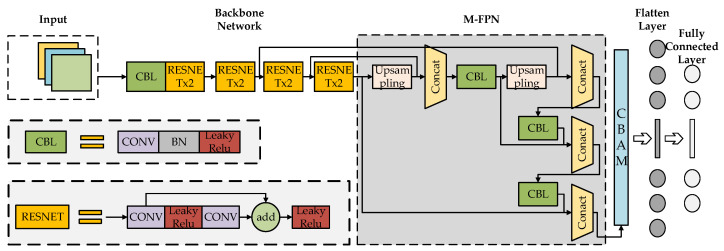
Improved ResNet18 network architecture.

For the problem of high-resolution reconstruction of 2D temperature fields, this paper chose to use ResNet18 as the backbone network for feature extraction to design the network. The part responsible for feature extraction mainly consists of four ResNet × 2 residual modules, where each residual block consists of two ResNet cells, and the structure of ResNet cells is shown in the bottom left of Figure 1, below. The input features will first go into a 3 × 3 ordinary convolution, followed by a LeakyReLU activation with another 3 × 3 ordinary convolution in the trunk section, followed by the result of the initial convolution being summed here with the features in the trunk section, and after that going into a LeakyReLU activation. The CBL structure in the figure is a standard convolutional layer consisting of normal convolution plus regularization and the LeakyReLU activation function, which is mainly responsible for assisting feature extraction.

The activation function, LeakyReLU, is a variant of the Modified Linear Unit (ReLU), which introduces a small slope in the negative region, usually a small positive number. It overcomes the “neuron death” problem of ReLU: ReLU outputs 0 in the negative region, which can lead to some neurons never being activated. LeakyReLU solves this problem by introducing non-zero slopes, so that even negative regions have some activation. This allows the network to avoid the problem of vanishing gradients during training. The mathematical expression is given in the following Equation (1).

(1) $$f(x)=\{\begin{array}{l} x,\;if\;x>0\\ \alpha x,\;if\;x\leq 0 \end{array}$$

where α is the leakage factor, which usually takes a value between 0 and 1.

For the feature enhancement part, this paper designed the structure of FPN+PAN. The main idea of the FPN structure is to integrate the features from top-down and bottom-up multiple paths for the fusion of high and low features, while the PAN structure focuses on the horizontal fusion of the data information as well as the bottom-up multi-scale fusion. The combined use of these two structures gives the model a more comprehensive multi-scale feature fusion capability. In the output part of the feature enhancement structure, this paper adds a CBAM attention mechanism layer, followed by a network spreading layer and, finally, a fully connected layer.

### 2.1. Optimization of Attention Mechanisms

Deep learning algorithms typically use the same convolutional operations frequently when processing data, and this causes the model to focus only on information that is localized to the data, resulting in relatively homogeneous extracted features. In traditional network architectures, to make the model learn more comprehensive feature information than the local information encapsulated by the convolution kernel, it is common to deepen the model depth of the network. After the local features are aggregated, the global features of the input information are further acquired. However, this approach tends to introduce a large number of model parameters, making the computation increase significantly.

With the addition of the attention mechanism, the model can not only utilize the parameters more effectively, but also focus on important parts of the data in a more targeted manner. By introducing the attention mechanism, the model can dynamically adjust the degree of attention to different regions during the learning process, thus reducing unnecessary computational overhead and improving the performance and efficiency of the model. This approach can effectively reduce computational costs while maintaining model accuracy, making deep learning models more efficient and flexible in processing data.

For the temperature field reconstruction task addressed by the text, we deeply customized and improved the spatial attention mechanism in the Convolutional Block Attention Module (CBAM) to enhance its effectiveness in the temperature field reconstruction task.

CBAM’s spatial attention mechanism generates two descriptive feature maps by performing average pooling and maximum pooling for each channel of the feature map, which are merged and passed through a convolutional layer to generate the final spatial attention map. This attention map is a single-channel map, and its higher values mark the regions to which the model should pay more attention.

**Figure 2:**
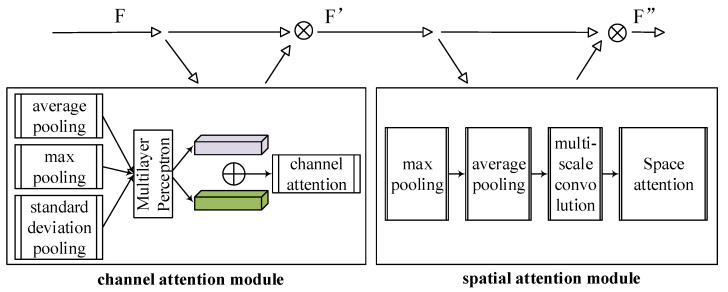
Improved CBAM spatial attention mechanism.

For the special needs of temperature field reconstruction, we realize that the traditional CBAM spatial attention mechanism may not be sensitive enough, especially when dealing with complex spatial variations and local temperature gradients that are common in temperature fields. Therefore, we improved the mechanism in the following points, and its structure is shown in Figure 2.

#### 2.1.1. Advanced Feature Aggregation

Diversified handling of feature aggregation methods is added, using not only the traditional mean pooling and maximum pooling, but also introducing standard deviation pooling to capture the variation information in the feature map. For the input feature map F with dimensions H×W×C, advanced feature aggregation is performed, including average pooling, maximum pooling, and standard deviation pooling. These three pooling operations provide global statistical information from different perspectives:

Average pooling:

(2) $$F_{\mathrm{avg}}(c)=\frac{1}{H\times W}\sum_{i=1}^{H}\sum_{j=1}^{W}F(i,j,c)$$

Maximum pooling:

(3) $$F_{max}(c)=maxF(i,j,c)$$

Standard deviation pooling (math.):

(4) $$F_{\text{std}}(c)=\sqrt{\frac{1}{H\times W}\sum_{i=1}^{H}\sum_{j=1}^{W}{\left(F(i,j,c)-F_{\text{avg}}(c)\right)}^{2}}$$

These pooling results are merged into a single feature descriptor, $F_{agg}$:

(5) $$F_{\mathrm{agg}}=\mathrm{Concat}(F_{\mathrm{avg}},F_{\max},F_{\mathrm{std}})$$

#### 2.1.2. Multi-Scale Convolution Processing

In order to enhance the model’s adaptability to structures of different scales, multi-scale convolutional processing is added before the convolutional layer that generates the spatial attention map. By using convolution kernels of different sizes (e.g., 1 × 1, 3 × 3, and 5 × 5), the model can take into account feature information ranging from small to large scales when generating the attention map.

Using multi-scale convolutional kernels, $F_{agg}$ convolutional operations are performed to capture spatial features at different scales:

(6) $$S=\sigma ({\text{Conv}}_{1\times 1}(F_{\text{agg}})+{\text{Conv}}_{3\times 3}(F_{\text{agg}})+{\text{Conv}}_{5\times 5}(F_{\text{agg}}))$$

where σ is the sigmoid activation function used to generate the final spatial attention graph, S. Each element of this graph has a value between 0 and 1, indicating the importance weight of the corresponding position.

#### 2.1.3. Dynamic Threshold Adjustment

After generating the final spatial attention maps, a dynamic threshold adjustment strategy was introduced to allow the model to automatically adjust the attention threshold based on the characteristics of different data. This allows the model to focus more flexibly and accurately on key regions when confronted with temperature field data with more subtle or complex features.

#### 2.1.4. Attention-Supervised Learning

In order to ensure the effectiveness of the spatial attention mechanism, attentional supervised learning is introduced to directly optimize the generation of the attention graph by means of a specialized loss function (e.g., the aforementioned attentional bootstrap loss).

(7) $$L_{\mathrm{attention}}=-\sum [M\log\left(S\right)+(1-M)\log\left(1-S\right)]$$

where M is the manually labeled critical area mask.

With these improvements, the performance of CBAM’s spatial attention mechanism in the temperature field reconstruction task is significantly enhanced, especially when dealing with complex spatial variations and localized temperature gradients.

### 2.2. Feature Pyramid Optimization

The role of a feature pyramid network is to provide a multi-scale feature map to better capture information at different scales in tasks such as feature extraction. Higher-level features in a feature pyramid network can contain more abstract and global information, while lower-level features contain more local and detailed information, which allows the network to reuse features at different levels and improve the efficiency of parameter utilization.

The feature pyramid network M-FPN with FPN+PAN structure designed in this paper fully utilizes the advantages of both. First, FPN realizes multi-scale information transfer through top-down feature fusion, which enables the model to better capture target information at different scales, thus improving the accuracy of detection. And PAN introduces a path aggregation mechanism, which can effectively aggregate features at different levels, further enhancing the model’s ability to perceive multi-scale information and helping to improve the accuracy of the semantic segmentation task. In addition, another advantage of the M-FPN structure is its higher computational efficiency. By reasonably combining and utilizing the features, unnecessary computational overhead is avoided and the parametric efficiency of the model is improved.

**Figure 3:**
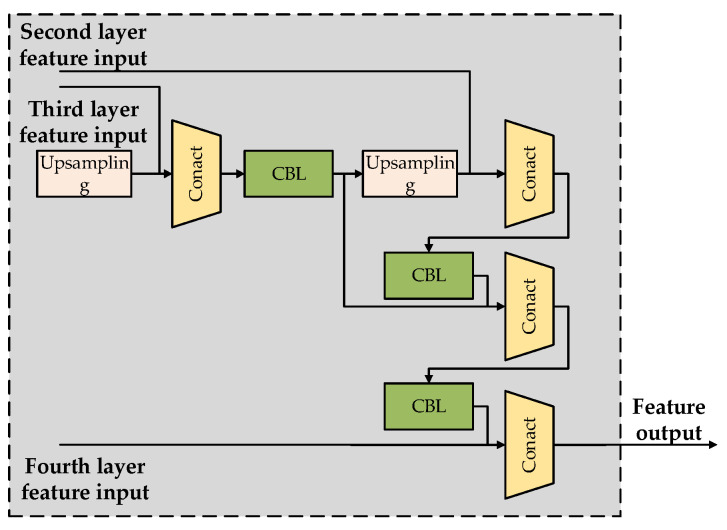
Structure of the feature pyramid.

The feature pyramid model M-FPN designed in this paper is structured as follows (Figure 3):

The main structure of the feature pyramid designed in this paper consists of four concat feature dimension splicing operations, which make full use of the shallow information extracted from the ResNet structural blocks in the previous section. By concatenating features from different layers in the channel dimension, the fusion of multi-scale information is realized, which improves the perceptual ability of the model pairs. This feature fusion approach can effectively synthesize the features from different layers, enabling the model to focus on both the details and the overall information of the data, thus better adapting to the number of feature inputs of different scales and complexities. Meanwhile, fusing the feature information together through the concat operation reduces the information loss and can better convey the gradient, which is conducive to the training and optimization of the model.

### 2.3. Loss Function Optimization

A suitable loss function can better guide the optimization process of neural networks and improve the training efficiency and performance of the model. Mean square error (MSE), as the most frequently used loss function in regression tasks, has the property of being continuously differentiable, which is suitable for gradient-based optimization algorithms such as gradient descent. It also has good mathematical convexity, which means that it has only one global minimum in the parameter space, and such a property makes it easier to find the optimal solution when using the MSE loss function. The penalty of the squared term makes the MSE more sensitive to large errors between predicted and true values, which helps the model to focus more on improving relatively poor predictions. Therefore, in this paper, the mean square error is used as the network loss function with the following formula:

(8) $$l_{MSE}=\frac{1}{J}\sum_{i=1}^{J}{\left(T_{i}-{\hat{T}}_{i}\right)}^{2}$$

where $l_{MSE}$ is the mean square error loss function, i.e., the mean square error between the predicted temperature value $T_{i}$ and the real temperature value ${\hat{T}}_{i}$ determined by the reconstruction algorithm, and the smaller value of $l_{MSE}$ represents the higher reconstruction accuracy. J is the number of fine-grid dissecting cells, and J = 400.

## 3. Simulation Verification

In order to verify the superiority of this paper’s algorithm compared with the original algorithm and the effectiveness of each improved module, firstly, single-peak, double-peak, and quadruple-peak Gaussian temperature field models were generated for the measurement area; then, the average relative error and the root-mean-square error were used as the evaluation indexes for assessing the quality of the temperature field reconstruction; finally, ablation experiments for the improved modules were designed, and the experimental results were analyzed.

### 3.1. Temperature Field Dataset Construction

**Figure 4:**
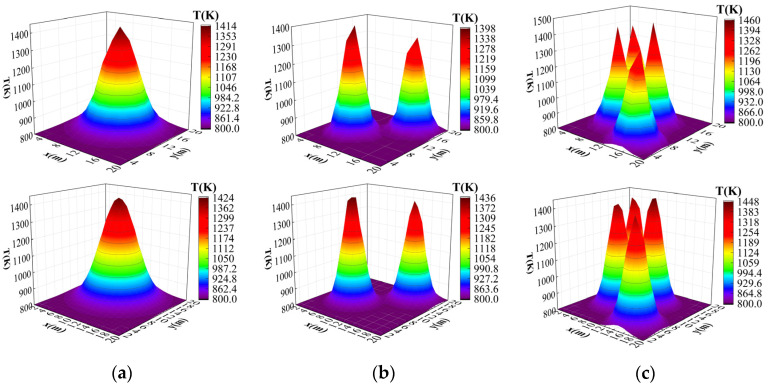
Example data from training and testing sets. (**a**) Example of a single-peak dataset. (**b**) Example of a bimodal dataset. (**c**) Example of a four-peak dataset.

The single-peak, double-peak, and quadruple-peak Gaussian temperature field models used in this paper were Equation (9), Equation (10), and Equation (11), respectively, and the temperature values of an arbitrary grid point (*x*, y on the 20 m × 20 m measurement area were as follows:

(9) $$T=T_{1}\cdot e^{-a\cdot [{\left(x-x_{1}\right)}^{2}+{\left(y-y_{1}\right)}^{2}]}+800$$

(10) $$T=T_{1}\cdot e^{-a\cdot \left({\left(x-x_{1}\right)}^{2}+{\left(y-y_{1}\right)}^{2}\right)}+T_{2}\cdot e^{-a\cdot \left({\left(x-x_{2}\right)}^{2}+{\left(y-y_{2}\right)}^{2}\right)}+800$$

(11) $$\begin{array}{l} T=T_{1}\cdot e^{-a\cdot \left({\left(x-x_{1}\right)}^{2}+{\left(y-y_{1}\right)}^{2}\right)}+T_{2}\cdot e^{-a\cdot \left({\left(x-x_{2}\right)}^{2}+{\left(y-y_{2}\right)}^{2}\right)}+\\ T_{3}\cdot e^{-a\cdot \left({\left(x-x_{3}\right)}^{2}+{\left(y-y_{3}\right)}^{2}\right)}+T_{4}\cdot e^{-a\cdot \left({\left(x-x_{4}\right)}^{2}+{\left(y-y_{4}\right)}^{2}\right)}+800 \end{array}$$

where $T_{1}$, $T_{2}$, $T_{3}$, $T_{4}$ is a random number within [600,700], a is randomly selected within [0.2,0.4], and $\left\{(x_{i}\;,y_{i\;})|i=1,2,3,4\right\}$ is associated with the peak position and is randomly generated within the grid. A total of 9000 pairs of coarse-grid and fine-grid temperature field data were randomly generated from Equation (9) to Equation (11) with 3000 pairs for each peak shape. The dataset was divided into training and test sets in the ratio of 9:1. Part of the data set is shown in Figure 4.

### 3.2. Network Training

In order to ensure the reproducibility of the experiments and the validity of the comparisons, all the experiments in this paper were carried out in identical environments. On the Windows system, PyCharm, an integrated development environment for the Python language, was used to build the Pytorch deep learning framework and test the improved algorithms in this paper. The relevant experimental environment configurations of this paper are shown in Table 1.

### 3.3. Network Performance Evaluation Metrics

In order to objectively evaluate the performance and effect of the model, the average relative error, $E_{ARE}$, and the root mean square error, $E_{RMSE}$, were used as the evaluation indexes to quantify the reconstruction effect, and a smaller value represents a better reconstruction effect of the temperature field, i.e.,

(12) $$E_{ARE}=\frac{1}{J}\sum_{i=1}^{J}\left|\frac{\hat{T}(i)-T(i)}{\hat{T}(i)}\right|$$

(13) $$E_{RMSE}=\frac{\sqrt{\frac{1}{J}\sum_{i=1}^{J}{\left(\hat{T}(i)-T(i)\right)}^{2}}}{{\hat{T}}_{\mathrm{mean}}}$$

where $\hat{T}$ and T are the model temperature distribution and the reconstructed temperature field, respectively; J is the number of grids for profiling; and ${\hat{T}}_{mean}$ is the average temperature value of the model.

### 3.4. Simulation Experiment

#### 3.4.1. Single-Peak Reconstruction Results

**Figure 5:**
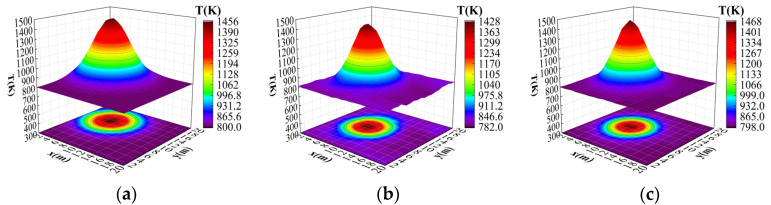
Results of single-peak temperature field reconstruction. (**a**) Original temperature field. (**b**) Original ResNet18 reconstructed temperature field. (**c**) Improved ResNet18 reconstructed temperature field.

In order to verify the effectiveness of the improved ResNet18 reconstruction algorithm presented in this paper, the reconstruction quality of the improved algorithm was initially verified using a single-peak Gaussian temperature field model, in which the parameters of the single-peak Gaussian temperature field model were $T_{1}$ = 609.43, $x_{1}$ = 8.24, $y_{1}$ = 11.62, and a = 0.3. Figure 5a–c show the original temperature field, the temperature field reconstructed by the original ResNet18 algorithm, and the temperature field reconstructed by the improved ResNet18 algorithm presented in this paper, respectively.

**Figure 6:**
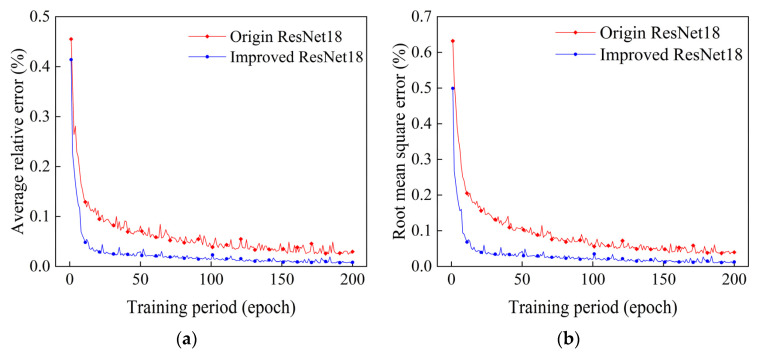
Plots of the relative errors in the reconstruction of single-peak temperature fields for different training periods. (**a**) Plot of mean relative errors. (**b**) Plot of root mean square errors.

After training the original ResNet18 reconstruction algorithm and the improved ResNet18 algorithm presented in this paper for 200 epochs, the trends of their reconstructed average relative errors and root mean square errors with training cycles were as shown in Figure 6a,b.

In this paper, the improved ResNet18 algorithm had an average relative error, $E_{\mathrm{ARE}}$, and root mean square error, $E_{RMSE}$, of 0.008% and 0.013%, respectively, after 200 epochs of training. The average relative error, $E_{\mathrm{ARE}}$, and the root mean square error, $E_{RMSE}$, of the original ResNet18 algorithm were 0.03% and 0.04%, respectively, after 200 epochs of training, which shows that the average relative error of the temperature field reconstructed by the improved algorithm of this paper was smaller for each grid compared with the original algorithm. Regarding the visual efficacy, both at the peak and at the edge, this paper’s improved algorithm has less distortion than the original algorithm, especially at the bottom of the single peak; it is capable of much smoother reconstruction than the original ResNet18 algorithm; and through the two-dimensional surface map, it can also be found that it has a smoother and more natural color in the adjacent region of the temperature field. Therefore, the improved ResNet18 algorithm presented in this paper does have better reconstruction efficacy compared with the original ResNet18 algorithm.

#### 3.4.2. Single-Peak Ablation Experiments

In order to individually verify the effectiveness of CBAM and M-FPN in the original ResNet18 network, this paper designed ablation experiments which contained four groups of experiments, where Group 1 was the original ResNet18 network model, Group 2 was the original ResNet18 network model to which the CBAM attentional mechanism was added alone, Group 3 was the original ResNet18 network model to which the M-FPN structure was added alone, and Group 4 was the original ResNet18 network model with both the CBAM attention mechanism and the M-FPN structure added. The results of the ablation experiments for each improvement point are shown in Table 2.

As can be seen from Table 2, the introduction of the CBAM attention mechanism reduced the average relative error and the root mean square error of the temperature field reconstruction by 0.01% and 0.012%, respectively, compared to the original ResNet18 network; this was because ResNet18+CBAM can dynamically adjust the degree of attention to the temperature of different regions during the reconstruction of the temperature field and pay more attention to important features in the temperature field and suppress irrelevant information, thus improving the utilization of the temperature data. After the introduction of M-FPN, the average relative error, $E_{\mathrm{ARE}}$, and the root mean square error, $E_{RMSE}$, of the temperature field reconstruction were also reduced compared with the original ResNet18 network, these values being 0.016% and 0.023%, respectively; this was because ResNet18+M-FPN can better fuse the data features of different network layers, realize the deep fusion of shallow and deep features, and increase the utilization rate of the model on multi-layer temperature data, thus improving the temperature field reconstruction effect. Meanwhile, after the introduction of the CBAM attention mechanism and M-FPN, the average relative error of the network, $E_{\mathrm{ARE}}$, was 0.008%, and the root mean square error, $E_{RMSE}$, was 0.013%; these values were thus reduced by 0.022% and 0.027%, respectively, compared with the original ResNet18 network model. ResNet18+CBAM+M-FPN collects the advantages of both and increases the utilization of multi-layer temperature information under the condition of guaranteeing full attention to important temperature information. It can be seen that the reconstruction accuracy of the temperature field after stacking two improved modules at the same time is better than that of the single module, and the M-FPN improved module has a greater temperature field reconstruction accuracy.

**Figure 7:**
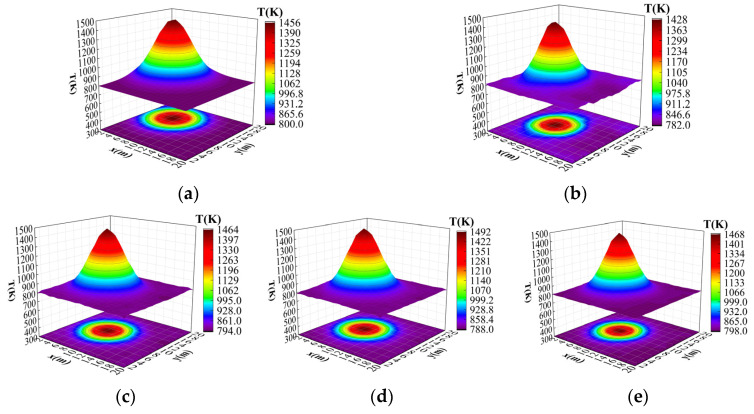
Results of single-peak temperature field reconstruction ablation experiments. (**a**) Original temperature field. (**b**) Original ResNet18 reconstructed temperature field. (**c**) Improved ResNet18 reconstructed temperature field. (**d**) Res-Net18+M-FPN. (**e**) ResNet18+CBAM+M-FPN.

Through the introduction of two improved methods (the CBAM attention mechanism and M-FPN multi-scale feature fusion), a comparison experiment with the original method was conducted, and the results show that the improved method proposed in this paper is significantly better than the original ResNet18 model in terms of reconstruction error. This comparison experiment fully verifies the effectiveness and robustness of this paper’s method in improving the accuracy and effect of temperature field reconstruction. Figure 7 demonstrates the specific impact of each improvement module on the reconstruction results in the ablation experiment, which further visualizes the enhancement of the reconstruction performance by each improvement.

#### 3.4.3. Bimodal Reconstruction Results

**Figure 8:**
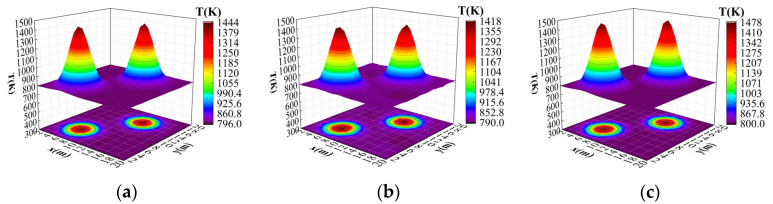
Results of bimodal temperature field reconstruction. (**a**) Original temperature field. (**b**) Original ResNet18 reconstructed temperature field. (**c**) Improved ResNet18 reconstructed temperature field.

In order to further verify the effectiveness of the improved algorithm presented in this paper, the bimodal Gaussian temperature field model was reconstructed, where the parameters of the bimodal Gaussian temperature field model were $T_{1}$ = 670.56, $T_{2}$ = 676.59, $x_{1}$ = 6.25, $y_{1}$ = 5.98, $x_{2}$ = 15.54, $y_{2}$ = 13.62, and a = 0.25, respectively. Figure 8a–c show the original temperature field, the temperature field reconstructed by the original ResNet18 algorithm, and the temperature field reconstructed by the improved ResNet18 algorithm presented in this paper, respectively.

**Figure 9:**
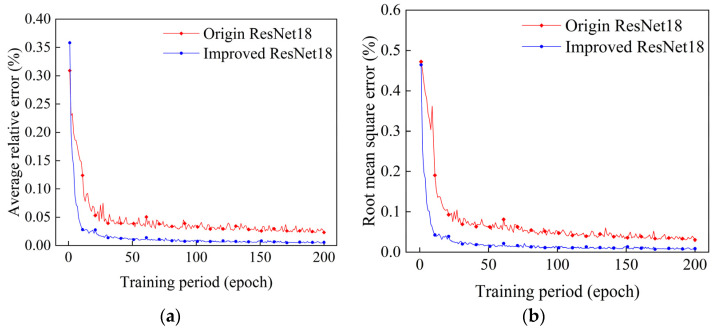
Plots of the relative errors in the reconstruction of bimodal temperature fields for different training cycles. (**a**) Plot of mean relative errors. (**b**) Plot of root mean square errors.

After training the original ResNet18 reconstruction algorithm and the improved ResNet18 algorithm presented in this paper for 200 epochs, the trends of their reconstructed average relative errors and root mean square errors with training cycles were as shown in Figure 9a,b.

After 200 epochs of training, the average relative error and the root mean square error of the improved ResNet18 algorithm were reduced by 0.018% and 0.021%, respectively, compared with the original ResNet18 algorithm; it can be seen that the average relative error for the temperature data in each grid reconstructed by the improved algorithm presented in this paper was smaller. Figure 8c shows more clarity in the peak region, and the reconstructed temperature distribution was closer to the real value, while Figure 8b shows some peak errors. In terms of edge processing, Figure 8c shows a better smooth transition, while Figure 8b shows some distortion. On the whole, Figure 8c shows less error than Figure 8b, especially in the high-temperature region and the edge region, which shows more stable performance. It shows that the improved ResNet18 algorithm presented in this paper has strong adaptability.

#### 3.4.4. Bimodal Ablation Experiments

The results of the ablation experiments at each improvement point designed in this paper for the bimodal Gaussian temperature field reconstruction are shown in Table 3.

As shown in Table 3, the average relative error and the root mean square error of the original ResNet18 were reduced after adding the CBAM module and the M-FPN module, respectively, but the reduction was limited. After adding the CBAM and M-FPN modules at the same time, the average relative error and the root mean square error reached the lowest values of 0.005% and 0.009%. It can be seen that the addition of the CBAM and M-FPN modules can indeed improve the temperature field reconstruction accuracy.

**Figure 10:**
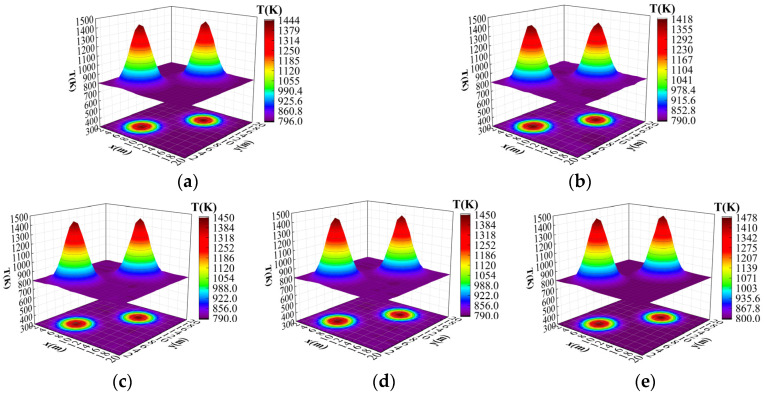
Results of bimodal temperature field reconstruction ablation experiments. (**a**) Original temperature field. (**b**) Original ResNet18 reconstructed temperature field. (**c**) Improved ResNet18 reconstructed temperature field. (**d**) Res-Net18+M-FPN. (**e**) ResNet18+CBAM+M-FPN.

Figure 10 demonstrates the specific impact of each improvement module on the reconstruction results in the ablation experiment, which further visualizes the enhancement of the reconstruction performance by each improvement.

#### 3.4.5. Four-Peak Reconstruction Results

**Figure 11:**
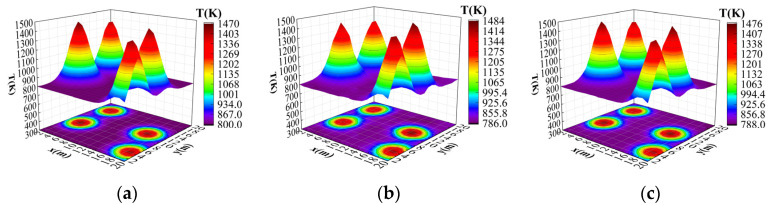
Results of four-peak temperature field reconstruction. (**a**) Original temperature field. (**b**) Original ResNet18 reconstructed temperature field. (**c**) Improved ResNet18 reconstructed temperature field.

In order to further verify the adaptability of the improved algorithm presented in this paper and to simulate more complex and variable real-temperature field distributions, a four-peak Gaussian temperature field model was used for reconstruction. The parameters of the example four-peak Gaussian temperature field model were $T_{1}$ = 632.24, $T_{2}$ = 599.40, $T_{3}$ = 653.09, $T_{4}$ = 632.242, $x_{1}$ = 3.72, $y_{1}$ = 8.52, $x_{2}$ = 4.46, $y_{2}$ = 16.26, $x_{3}$ = 16.23, $y_{3}$ = 112.41, $x_{4}$ = 18.83, $y_{4}$ = 3.89, and a = 0.21. Figure 11a–c show the effect of the original temperature field, the temperature field reconstructed by the original ResNet18 algorithm, and the temperature field reconstructed by the improved ResNet18 algorithm presented in this paper, respectively.

**Figure 12:**
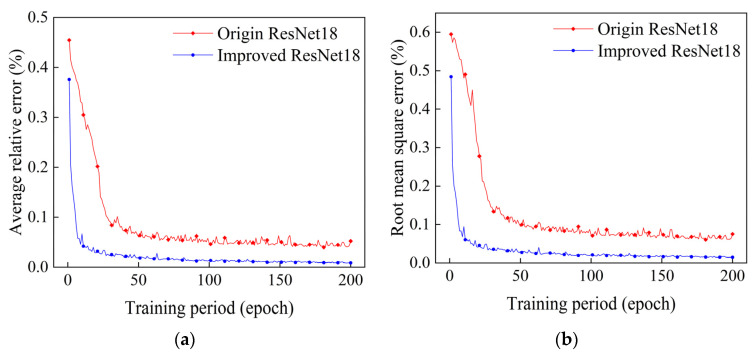
Plots of the relative errors of the reconstruction of four-peak temperature fields with different training cycles. (**a**) Plot of mean relative errors. (**b**) Plot of root mean square errors.

After training the original ResNet18 reconstruction algorithm and the improved ResNet18 algorithm presented in this paper for 200 epochs, the trends of their reconstructed average relative errors and root mean square errors with training cycles were as shown in Figure 12a,b.

As can be seen in Figure 12, similar to the single-peak and double-peak Gaussian temperature field reconstruction results, the reconstruction effect of this paper’s improved ResNet18 algorithm on the four-peak temperature field was still better than that of the original ResNet18 algorithm, and the average relative error and the root mean square error were reduced by 0.043% and 0.06%, respectively, after 200 epochs of training. Figure 8 also shows that the reconstruction results of the improved algorithm presented in this paper are more similar to the original temperature field model than those of the original ResNet18 algorithm in terms of the distribution location of peaks, the amplitude of temperature, and the trend of temperature data. This fully demonstrates that the improved ResNet18 algorithm presented in this paper can still maintain high stability even when four-peak complex temperature field reconstruction is performed.

#### 3.4.6. Four-Peak Ablation Experiments

In this paper, for the four-peak Gaussian temperature field reconstruction, the results of the ablation experiments for each designed improvement point are shown in Table 4.

As can be seen, similar to the single-peak and double-peak reconstruction results, the improved method presented in this paper achieved the best reconstruction quality, and its average relative error and root mean square error were the lowest, being 0.009% and 0.015%, respectively. Although the reconstruction effect was significantly improved after introducing the CBAM module and the M-FPN module alone, the reconstruction effect was still lower than that of the ResNet18+CBAM+M-FPN algorithm described in this paper, which fully proves the effectiveness of the improved module.

**Figure 13:**
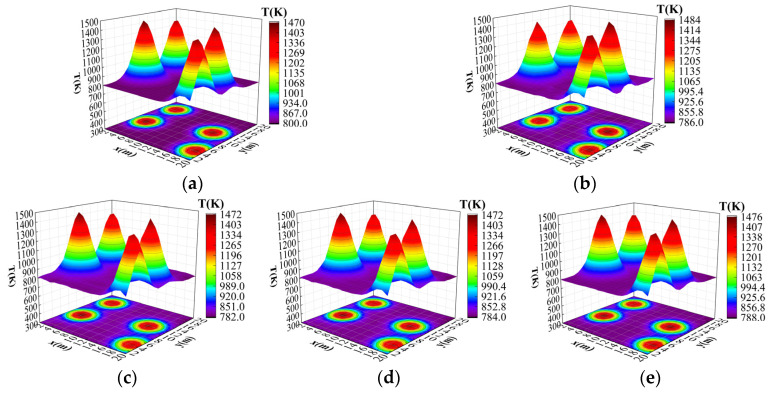
Results of four-peak temperature field reconstruction ablation experiments. (**a**) Original temperature field. (**b**) Original ResNet18 reconstructed temperature field. (**c**) Improved ResNet18 reconstructed temperature field. (**d**) Res-Net18+M-FPN. (**e**) ResNet18+CBAM+M-FPN.

Figure 13 demonstrates the specific impact of each improvement module on the reconstruction results in the ablation experiment, which further visualizes the enhancement of the reconstruction performance by each improvement.

## 4. Conclusions

(1) Focusing on the problem of the low accuracy of the original ResNet18 network model for high-resolution reconstruction of temperature fields, this paper proposes an improved ResNet18 algorithmic model for temperature field reconstruction, which introduces the CBAM attention mechanism to strengthen the attention to important information and the multi-scale feature pyramid M-FPN to improve the utilization of multi-layer information on the basis of the original ResNet18.
(2) In order to verify the effectiveness and stability of the algorithm, multiple Gaussian temperature distribution models are generated by simulation, and the reconstruction results show that the improved ResNet18 method in this paper can obtain higher reconstruction quality.
(3) In this paper, the effects of the CBAM attention mechanism and the multi-scale feature pyramid M-FPN on the temperature field reconstruction accuracy are analyzed by ablation experiments. The results show that for single-peak, double-peak, and four-peak temperature field reconstruction, either introducing the CBAM module alone or the M-FPN module alone, reconstruction quality improvement can be obtained, and the best reconstruction quality with a smaller reconstruction error is obtained after the double-improvement module is introduced at the same time, and the reconstruction effect of the proposed method in this paper is better than the original algorithm.
(4) Despite these improvements, some limitations of the proposed method should be acknowledged. The increased complexity of the model due to the inclusion of both the CBAM and M-FPN modules leads to longer training times and higher computational costs. Moreover, while the method demonstrates strong performance across a variety of test cases, its effectiveness in more extreme or highly complex temperature fields may still require further investigation. Future work will focus on optimizing the computational efficiency of the model, exploring lighter architectures, and investigating the method’s scalability to more diverse industrial scenarios. Additionally, potential improvements such as integrating more advanced data augmentation techniques or combining this approach with reinforcement learning could further enhance the model’s adaptability and generalization capabilities in real-world applications.

## Figures and Tables

**Table 1:** Experimental environment configuration table.

Configuration	Configuration Parameter
Operating system	Windows 11 64-bit
CPU	12th Gen Intel(R) Core(TM) i5-12490F
GPU	NVIDIA GeForce RTX 3060 (12 GB)
Memory	32 GB
Deep learning framework	Pytorch 1.7.1+ Cuda11.0
Optimizer	SGD
Initial learning rate	0.01
Batch size	32
Epochs	200

**Table 2:** Experimental results of ablation at different improvement points for single-peak Gaussian temperature fields.

Method	E_ARE_/%	E_RMSE_/%
ResNet18	0.03	0.04
ResNet18+CBAM	0.02	0.028
ResNet18+M-FPN	0.014	0.017
ResNet18+CBAM+M-FPN	0.008	0.013

**Table 3:** Experimental results of ablation at different improvement points of bimodal Gaussian temperature field.

Method	E_ARE_/%	E_RMSE_/%
ResNet18	0.023	0.030
ResNet18+CBAM	0.015	0.021
ResNet18+M-FPN	0.012	0.018
ResNet18+CBAM+M-FPN	0.005	0.009

**Table 4:** Experimental results of ablation at different improvement points of four-peak Gaussian temperature field.

Method	E_ARE_/%	E_RMSE_/%
ResNet18	0.052	0.075
ResNet18+CBAM	0.036	0.048
ResNet18+M-FPN	0.030	0.039
ResNet18+CBAM+M-FPN	0.009	0.015

## Data Availability

Results can be provided upon request.
